# Scales of risk and adaptive ‘dread’: an evolutionary theory of risk inflation

**DOI:** 10.1038/s41598-025-19079-3

**Published:** 2025-11-10

**Authors:** John M. McNamara, Sasha R. X. Dall, Alasdair I. Houston

**Affiliations:** 1 School of Mathematics, University of Bristol, Bristol, UK; 2 Centre for Ecology and Conservation, University of Exeter, Penryn, UK; 3 School of Biological Sciences, University of Bristol, Bristol, UK

**Keywords:** Fluctuating environment, Environmental (aggregate) risk, Mortality, Human behaviour, Evolutionary theory

## Abstract

People often react to low probability, high damage events in which many die with strong avoidance behaviour. Indeed, analyses of behaviour following the September 11 terror attacks on New York City suggest that this caused a substantial number of additional, ‘indirect’ deaths as many people avoided flying for 12 months afterwards and took to the relatively risky highways of the US instead. We argue that such responses may have arisen as an adaptation to risks that wipe out a significant proportion of all carriers of an allele if they strike, e.g. storms. These are environmental fluctuations known as environmental or aggregate risks. At the opposite extreme, demographic risks affect individuals independently. We show that evolution by natural selection in fluctuating environments means it is adaptive to inflate environmental (aggregate) risks relative to demographic risks, where the inflation factor depends on the proportion of carriers of the allele that die if the risk strikes.

## Introduction

The world is a risky place, full of uncertain hazards. It probably always has been over the evolutionary history of most animals^1^, even if some of the most serious risks, as manifest in per capita mortality rates, appear to have declined sharply, and continue to decline, for modern humans^2,3^. Perception of risk and the measurable likelihood of harm to humans are not always correlated^4,5^. Nuclear power is viewed by experts as relatively safe but non-experts rate it as very risky^6^, an assessment that is likely to impose a cost in terms of supplying energy^7^. Similar remarks apply to public fears about terrorism^8^, which can generate substantial economic overinvestment into mitigating such risks at the expense of other, more measurably pervasive societal needs (like education or healthcare). After the attacks on the New York World Trade Center in 2001, US government spending on homeland security increased dramatically^9^, despite one of the most catastrophic contractions in US (and global) economic output on record in 2008.

It is well documented that people often respond to uncertain hazards that are perceived as involuntary, uncontrollable, lethal or unfair with strong aversion to situations associated with such risks, especially if they impact many people at once (e.g.^6,10^). Thus, they are often referred to as ”dread risks”^6,9,11–14^, and the more unknown, unfamiliar and systemic their causes are, the more people behave as if circumstances associated with such hazards predict future risk^10^.

It is perhaps unsurprising then that terror as an insurgency tactic seems to exploit the psychological characteristics of dread. Indeed, an influential analysis of people’s behaviour in the USA after the September 11th 2001 attacks^11,12,15^ provides a striking illustration of how substantial the dread risk response to terror can be. There is strong evidence that travelling by car is much more dangerous than travelling by plane^16–18^. Gigerenzer^11,12^ documented that Americans avoided flying after 9/11 and many drove instead, which resulted in an estimated 1,595 additional road deaths that can be considered ”indirect terrorism damage” caused by a dread response to the attacks^12^. He interpreted this response as maladaptive, caused by a failure to accurately assess the relative risks associated with the different long-distance travel options available, and called for a concerted effort to inform people about such risks as part of a psychologically cognisant ”extended counterterrorism policy”^12^. Although preferring to drive is not rational in the current environment, we show here how such a response, even when fully-informed of the relative risks involved, could be adaptive in a particular environment if the evolutionary consequences of mortality risks at different scales are properly accounted for.

There has been little theoretical effort to elucidate the evolutionary origins of dread risk responses. The most well-developed ideas so far have focussed on the adaptive value of dread responses to perceived increases in mass-casualty risks. These have proposed that the kind of dread bias behaviour documented for 9/11 might have been selected for in our ancestors. For example, Bodemer et al.^13^ analyse dread in terms of the influence of mortality events on a measure of cumulative population size. We agree with Bodemer et al. that organisms should evolve so that dying from some sources of mortality is regarded as worse than dying from others. However, their model has no genetic component and so fails to capture the essence of the issue (see “Discussion”). Natural selection acts on gene frequencies, and from this perspective individuals are only important in so much as they transmit genes to future generations^19–21^. Some sources of mortality affect individuals carrying a specific gene independently of other carriers (demographic, or idiosyncratic, risk), other sources wipe out a significant proportion of all carriers of the genes if they strike (environmental, or aggregate, risk)^22,23^. Here we show formally how substantial biases against the latter can evolve, even when they expose individuals to higher levels of demographic risk, and thus may explain dread responses of the sort associated with mass-casualty terror attacks.

### Model details

We suppose that the risk behaviour of an individual is an inherited trait with a genetic basis. Our aim is to predict the value of this trait that will evolve under the action of natural selection. To do so, we use the standard approach and assume that the trait is specified by the allele present at a single given autosomal locus^24,25^, while recognising that in reality risk behaviour is probably influenced by many genetic loci. Under our simplifying assumption, different alleles present at this given locus specify different risk behaviours. The behaviour specified by an allele affects the number of offspring left by the carriers of the allele, and hence the number of copies of the allele present in the next generation. In this way, the number of copies of an allele present in the population has a characteristic rate of change over generations. This rate of change is known as the invasion fitness of the allele. The action of natural selection is then predicted to result in a population in which most individuals carry the allele with the greatest invasion fitness. Thus at evolutionary stability we expect the evolved risk behaviour to maximise the invasion fitness of the allele that is responsible for this behaviour.

When we focus on a particular allele we make the simplifying assumption that all bearers of the allele have just a single copy. Thus the size of the cohort of individuals carrying the allele equals the total number of copies of the allele. The rate of increase in allele numbers (invasion fitness) then equals the rate of increase in cohort numbers. This rate of increase is affected by reproduction and mortality. All individuals reproduce continually: the rate at which they transmit the focal allele to offspring is *R*, irrespective of the allele’s effect on risk behaviour. Individuals experience two sources of mortality, where the level of mortality depends on risk behaviour and hence on the allele we are concerned with. One source of mortality is demographic; that is different cohort members are affected independently (idiosyncratic risk). The mortality rate per individual from this risk is $M_D$. The other source is environmental (aggregate risk). This is modelled by assuming that there is the occasional environmental catastrophe. These catastrophes occur as a Poisson process of rate $\lambda$. When a catastrophe occurs a fixed proportion, *p*, of the cohort die. Thus the mortality rate from catastrophes per individual carrying the focal allele is $M_C = \lambda p$.

In the above $\lambda$ is a fixed environmental parameter. In contrast, the parameters $M_D$ and *p* depend on risk behaviour, and hence on the focal allele.

## Results

We divide our results into two parts. We begin by deriving a formula for the invasion fitness of an allele that takes into account the two source of mortality associated with the allele. This formula highlights that the two sources of mortality are not equivalent. In the second part, we assume that individuals face a trade off between the two sources of mortality and analyse how we expect natural selection to shape risk attitudes under this trade off.

**The rate of increase in allele numbers and inflation.** In calculating the invasion fitness of an allele, we must take into account the two sources of stochasticity. Idiosyncratic risk acts independently on different carriers of the allele, so that we can just average over this source of stochasticity when the numbers of carries of the allele are large. In contrast, aggregate risk affects many carriers in the same way so that this simple form of averaging is not appropriate. This is well known in gambling and investment when the objective is to maximise the long-term growth in wealth. In these scenarios, even if a bet or investment has the highest average return currently available, if the bet is risky it is best to only allocate a fraction of current wealth to it in case one is unlucky. Here the Kelly criterion^26^ provides a money management strategy that maximises the geometric growth rate of wealth. Note that maximising the geometric growth rate is equivalent to maximising its logarithm. In considering invasion fitness, the long-term increase in allele numbers is analogous to the long-term growth in wealth. As we show in Methods (below), one should average over the logarithmic increase in numbers of an allele as the invasion fitness measure^24,27^. In Methods we show that invasion fitness is

1 $$\begin{aligned} r \ = \ R - (M_D + \alpha M_C), \end{aligned}$$

where

2 $$\begin{aligned} \alpha = -\frac{\log (1-p)}{p}. \end{aligned}$$

**Fig. 1:**
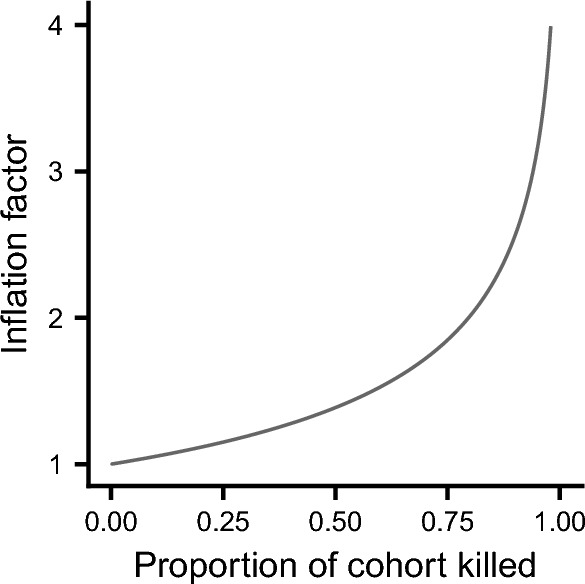
The inflation factor, $\alpha$, given by Eq. (2) as a function of the proportion of carriers of an allele that are killed in an environmental catastrophe.

These formulae demonstrate that the two sources of mortality are not equivalent in their effect on invasion fitness. Specifically, the risk from the environmental source of mortality is inflated by the factor $\alpha$ relative to the risk from the demographic source of mortality. The inflation factor is always greater than 1, and increases as the proportion of the cohort killed in a catastrophe, *p*, increases. Figure 1 shows the dependence of the inflation factor on *p*. Thus for example, if 60% of the cohort is killed in a catastrophe then the effect of this source of mortality on invasion fitness is inflated by around 50%. To illustrate this, suppose that catastrophes happen on average every 20 years and kill 60% of the cohort. Then the actual mortality rate from catastrophes would be 1 death per head of population every 30 years. But the catastrophes would reduce invasion fitness by approximately the same amount as a demographic source of mortality that caused one death per head of population every 20 years.

**Spreading the risk.** Now suppose that population members can decide which source of mortality to expose themselves to. Think of this as travelling individually by car versus travelling with others by plane. We assume that population members have to undertake a relatively safe journey every day or week. If they use a car then they die on the journey with probability $\mu$, independently of other population members. If they use the plane, the plane crashes with probability $\lambda$, and all individuals who use the plane that day die. For simplicity, we assume that there is no frequency dependence acting on these mortality rates. Specifically, the number of population members that choose to use a car, and hence the density of cars on the roads, does not affect the probability, $\mu$, that a given car crashes. Similarly, the numbers deciding to choose a plane does not affect whether a specific plane crashes.

We assume that the decision on the form of transport is determined by an allele which specifies the probability, *p*, that the bearer of the allele takes the plane on each journey. Whether a car or plane is chosen by one individual is independent of the choice of others. As before we are concerned with the cohort of individuals that carry this allele and consider the growth rate in the size of the cohort. Assuming reasonably large number of individuals (so that we can average over demographic stochasticity), the proportion of cohort members that use the plane on each journey is *p*, so that $M_C= \lambda p$ and $M_D=(1-p)\mu$. By Eqs. (1) and (2) the invasion fitness of the allele is

3 $$\begin{aligned} r(p)=R-(1-p)\mu + \lambda \log (1-p). \end{aligned}$$

Let $p^*$ be the value of *p* that maximises this fitness measure. Since there is no frequency dependence, the allele which codes for this probability of plane travel will spread to fixation in any resident population. Thus travelling by plane with this probability is the unique evolutionarily stable strategy.

Differentiating with respect to *p* we have

4 $$\begin{aligned} \frac{dr}{dp} = \mu - \frac{\lambda }{1-p}. \end{aligned}$$

Thus if $\lambda > \mu$ invasion fitness always decreases as *p* increases, so that $p^*=0$; i.e. at evolutionary stability everyone goes by car. However, if $\lambda < \mu$ at evolutionary stability a proportion

5 $$\begin{aligned} p^*=1- \frac{\lambda }{\mu } \end{aligned}$$

go by plane. Note that in this case the actual per capita mortality rate for the cohort due to individuals taking the car is $(1-p^*)\mu = \lambda$, while that from the plane is $p^* \lambda$. So no matter how safe the plane is compared with the car, more cohort members die in car accidents than in plane accidents. In the limit as the plane becomes ultra safe compared with the car ($\lambda \ll \mu$, so that $p^* \eqsim 1$), the numbers dying in plane crashes and motor accidents are almost equal.

## Discussion

Our approach envisages that attitudes to risk have a genetic basis, with the alleles at an autosomal locus specifying the attitude of the carrier of the allele. It is a standard argument that at an evolutionary endpoint, the allele that has been selected for should maximise its invasion fitness^24,28^. This means that the rate of growth in numbers of the allele is maximised. The appropriate measure of ”growth” is the geometric mean increase in numbers while the allele is rare, or the generalisation of this concept to structured populations^24,27^.

Risk factors range in the correlation of their effect on different individuals carrying the allele between two extremes. At one extreme, under a demographic (idiosyncratic) risk factor, such as bad luck in finding food, the death of an individual carrying the allele is independent of the deaths of other carriers. At the other extreme, under an environmental (aggregate) risk factor, such as bad weather, a significant proportion of all carriers of the allele die.

It has long been realised that the form of risk is crucial to the measure of invasion fitness^29^; see also^22,30–32^. Furthermore, this body of work stresses that environmental risk has a disproportionate effect compared with demographic risk. For instance, Robson^22^ observes that the fitness criterion ”incorporates an essential distinction between idiosyncratic risk given the environment and aggregate uncertainty concerning the environment itself, implying a greater aversion toward the latter. This is because the present model generates a form of automatic biological insurance via the law of large numbers against idiosyncratic risk, whereas this insurance is inoperative in the same sense against aggregate uncertainty”. This effect is illustrated in the context of financial investment by^33^.

One can rephrase these arguments to infer that if evolution leads to an individual being indifferent between a demographic risk and an environmental risk, then the probability of death under the environmental risk must be less. Our contribution in this paper is to quantify exactly how much less, with the inflation factor relating the probabilities of death under the two forms of risk just depending on the proportion of the carriers of the focal allele that die if the deleterious environmental event occurs.

When a new allele arises, then while it is still rare, if an individual carries the allele then others carrying the allele are kin. Thus the inflation effect should be seen if there was limited gene flow between groups of early humans. Limited gene flow means that almost all copies of the allele are within the group where it arose, so a risk that affects the whole group is an aggregate risk. In contrast, if there is substantial gene flow, then a risk that only affects one group acts more as a demographic risk. This contrast is illustrated in^23^ where there is a switch away from risk-averse behaviour as the dispersal rate between local groups increases. Data from current hunter gatherers suggests that there is significant movement of individuals between groups^34^, so at first sight, early humans may not have experienced limited gene flow. Even if early humans had the same social structure as current hunter gatherers this is not conclusive, however, because the analysis in^23^ assumes that the experience of each group is independent. If groups are not independent (e.g. because they experience the same weather conditions) dispersal will not eliminate aggregate risk because carriers of a focal gene in different groups will experience similar conditions.

In the modern world, many people prefer to drive rather than travel by plane. This avoidance of planes does not make evolutionary sense unless an individual is travelling with a substantial proportion of close kin. An individual not travelling in such circumstances should prefer the option that is safer. But in general, people do not assess probability of death as an actuary would; instead they use feelings which are likely to have been shaped by natural selection^35,36^. As a result they may be inaccurate in their estimates of risk^5,12,37^.

Like us, Bodemer et al.^13^ adopt an evolutionary approach and argue that dread is rational. Their analysis goes beyond ours in considering an age-structured population, but it has some serious limitations. One is that the measure they use (people-years) ignores population structure and depends on the time period over which it is measured. Rather than looking at the growth of the whole population (e.g. the whole of the USA), an evolutionary analysis should consider a cohort comprising those individuals that share an invading allele. In doing so we use geometric fitness because we are concerned with an aggregate risk that affects many individuals that carry a given gene. This crucial aspect of risk is absent from Bodemer et al., as is the frequency of the risk. In contrast, we have derived a simple expression which can be used to titrate the two forms of risk against one another, where the titration depends on the proportion of kin that are killed in an event that inflicts aggregate risk.

## Methods

We consider a rare mutant allele. We suppose that individuals that carry this allele each have a single copy of the allele. The behaviour of such an individual leads to a probability *p* that the individual will die should an environmental catastrophe occur. We follow the cohort of such individuals over time. Let *N*(*t*) be the number of cohort members present at time *t*, which equals the number of copies of the allele at time *t*. It is assumed that this number is large, so that we can average over demographic stochasticity. Then, between catastrophes the change in number is given by

6 $$\begin{aligned} \frac{dN}{dt} = RN(t) - M_D N(t). \end{aligned}$$

Let $x(t)= \log (N(t))$. Then between catastrophies

7 $$\begin{aligned} \frac{dx}{dt} = R-M_D. \end{aligned}$$

During a catastrophe there is a downward jump in *N*(*t*):

8 $$\begin{aligned} N(t) \rightarrow (1-p)N(t). \end{aligned}$$

Thus

9 $$\begin{aligned} x(t) \rightarrow x(t)+ \log (1-p). \end{aligned}$$

(Note that $\log (1-p)$ is negative.) The stochastic process $\{ x(t):t \ge 0 \}$ is time stationary. Since catastrophes occur at rate $\lambda$, by Eqs. (7) and (9) this process has mean drift

10 $$\begin{aligned} r=R-M_D+ \lambda \log (1-p). \end{aligned}$$

The long-term rate of increase in allele numbers is maximised by maximising this drift. We therefore take *r* to be the invasion fitness of the allele. [Note that $e^r$ is the geometric mean rate of increase in allele numbers (e.g.^27^)]. Using the relation $M_C= \lambda p$ we have

11 $$\begin{aligned} r&=R- M_D+ (M_C/p) \log (1-p)\end{aligned}$$

12 $$\begin{aligned}&= R - (M_D + \alpha M_C). \end{aligned}$$

## Data Availability

All data generated or analysed during this study are included in this published article.
